# Two Linked Enteroinvasive *Escherichia coli* Outbreaks, Nottingham, UK, June 2014

**DOI:** 10.3201/eid2207.152080

**Published:** 2016-07

**Authors:** Sophie Newitt, Vanessa MacGregor, Vivienne Robbins, Laura Bayliss, Marie Anne Chattaway, Tim Dallman, Derren Ready, Heather Aird, Richard Puleston, Jeremy Hawker

**Affiliations:** Public Health England, Nottingham, UK (S. Newitt, V. MacGregor, V. Robbins, L. Bayliss, R. Puleston);; Public Health England, London, UK (M. Chattaway, T. Dallman, D. Ready);; Public Health England, York, UK (H. Aird);; Public Health England, Birmingham, UK, and National Institute of Health Research, Liverpool, UK (J. Hawker)

**Keywords:** Enteroinvasive, *Escherichia*, *E. coli*, EIEC, EIEC O96:H19, bacteria, foodborne, gastrointestinal illness, Nottingham, England, United Kingdom, enteric infections

## Abstract

Enteroinvasive *Escherichia coli* (EIEC) outbreaks are uncommon in Europe. In June 2014, two EIEC outbreaks occurred in Nottingham, UK, within 2 days; outbreak A was linked to a takeaway restaurant and outbreak B to a wedding party. We conducted 2 analytical studies: a case–control study for outbreak A and a cohort study for outbreak B. We tested microbiological and environmental samples, including by using whole-genome sequencing. For both outbreaks combined, we identified 157 probable case-patients; 27 were laboratory-confirmed as EIEC O96:H19–positive. Combined epidemiologic, microbiological, and environmental findings implicated lettuce as the vehicle of infection in outbreak A, but the source of the organism remained unknown. Whole-genome sequencing identified the same organism in cases from both outbreaks, but no epidemiologic link was confirmed. These outbreaks highlight that EIEC has the capacity to cause large and severe gastrointestinal disease outbreaks and should be considered as a potential pathogen in foodborne outbreaks in Europe.

## Abstract 2

These outbreaks highlight the necessity to consider this bacterium as a potential pathogen in foodborne outbreaks.

Enteroinvasive *Escherichia coli* (EIEC) bacteria are human enteric pathogens that have been identified worldwide. EIEC has been found to be endemic to developing countries, particularly where sanitation is poor, and causes illness in both adults and children (*1*–*3*). EIEC are genetically similar to *Shigella*; both genera contain the *ipaH* invasive gene (*4*) and cause invasive disease that may result in severe illness in otherwise healthy persons (*5*). Transmission of EIEC is by the fecal–oral route, and contaminated food or water are the usual vehicles of infection.

EIEC outbreaks are rare in Europe; cases are typically sporadic and travel-related (*6*,*7*). EIEC outbreaks have been reported in Hungary in 1959 (*8*), Czechoslovakia in 1982 (*9*), and Israel in 1990 (*10*). The only recently reported EIEC outbreak in western Europe was in Italy in 2012 (*5*), and no outbreaks have been reported in the United Kingdom or other parts of northern Europe.

In June 2014, Public Health England (PHE) (East Midlands) was notified of 2 suspected gastroenteritis outbreaks within 2 days of each other. On June 26, 2014, PHE received a report of 7 patients admitted to an emergency department with diarrhea, vomiting, and fever 24 hours after consuming food purchased at a local takeaway restaurant in Nottingham (outbreak A). An outbreak control team was convened and Environmental Health Officers issued a Hygiene Emergency Prohibition Notice to close the restaurant. On June 27, 2014, PHE received a report of another outbreak of gastrointestinal illness characterized by diarrhea and vomiting after a wedding party on June 24 at a second restaurant in Nottingham (outbreak B), located within 0.1 miles of the restaurant implicated in outbreak A. Initial culture-based methods used to test the fecal specimens from both outbreaks had negative results for enteric organisms routinely tested for at the local laboratory; specimens were then sent to the Gastrointestinal Bacterial Reference Unit at PHE London (GBRU).

The 2 outbreaks were considered potentially linked in time, person, and place and were investigated to identify their potential sources. We report the findings of the investigations into these EIEC outbreaks.

## Methods

### Epidemiologic

We conducted 2 separate analytical epidemiologic studies to investigate the outbreaks: a case–control study with case-nominated controls for outbreak A, and a cohort study for outbreak B. We created 2 separate questionnaires for the outbreaks to collect data on basic demographics, symptoms and onset dates, contact with healthcare services, travel, contact with persons with diarrhea and vomiting in the 10 days before illness, and food consumed in each restaurant. PHE staff interviewed eligible study participants by telephone.

#### Outbreak A Investigation

A probable case-patient was defined as a person who consumed food from the restaurant during June 12–26, 2014, and within 7 days of exposure had diarrhea or >2 of the following symptoms: vomiting, nausea, abdominal pain, fever, muscle ache or influenza–like symptoms, or headache; and who had no history of travel abroad or contact with anyone who had diarrhea or vomiting during the 10 days before onset, whether or not PCR assay detected *ipaH* gene from a fecal sample. Confirmed case-patients were defined as above plus EIEC O96:H19 isolated from a fecal sample.

Cases were identified through laboratory surveillance, notifications from clinicians in healthcare settings, and calls to the environmental health team. Healthcare providers in the area were alerted to notify any persons with suspected cases of food poisoning who had recently eaten at the restaurants. Restaurant staff were investigated separately and excluded from the analytical study.

 The restaurant did not keep records of customers, so case-patients were asked to nominate controls by providing details of persons they knew who had eaten at the restaurant. A control was defined as a person who had consumed food from the restaurant during the same time period (June 12–26, 2014) but who did not have diarrhea, vomiting, nausea, abdominal pain, or fever and muscle ache or influenza–like symptoms since then.

#### Outbreak B Investigation

Case definitions for outbreak B were the same as for outbreak A, but case-patients consumed food at a wedding party, in a different restaurant from the one associated with outbreak A, on June 24. A list of persons who had attended the wedding was compiled by the Environmental Health Officers by consulting one of the wedding party organizers.

### Statistical Analyses

The sample size for both outbreaks was not calculated a priori, but was determined by the number of available case-patients and controls. We retrospectively calculated the power of the studies on the basis of the final sample size.

Descriptive analysis was undertaken for each outbreak by time, person, and place. Univariable analysis was undertaken to calculate odds ratios (case–control) and relative risks (cohort) with 95% CIs. Variables that had a p value <0.25 in the univariable analysis were included in the multivariable model. We conducted multivariable analysis using logistic regression (case–control) and Poisson regression with robust SEs (cohort), using a backward stepwise elimination process for both. We used Stata version 12 (StataCorp LP, College Station, TX) for analysis.

### Microbiological

Fecal samples from case-patients and food handlers were submitted to the GBRU for PCR testing for a range of pathogenic markers associated with *Shigella* spp. and the 5 diarrheagenic *E. coli* groups. Primers and conditions were as previously described, including the enteroinvasive ipaH gene associated with *Shigella* spp. and EIEC (*11*), the EAEC regulation gene aggR (*12*), the ETEC LT/ST toxin genes (*13*), Shiga toxin genes stx1 and stx2 for STEC, and the effacement and attachment gene eae for EPEC and the O157rfb gene (*14*). Additionally, the first 59 fecal samples underwent multiplex PCR testing for other bacterial and viral pathogens, as previously described at the regional laboratory (*15*).

We selected isolates from the outbreak for whole genome sequencing and phylogenetic analysis as described (*16*). Short reads were quality trimmed (*17*) and mapped to the Spades version 2.5.1 (*18*) de novo assembly of 1 EIEC genome isolated by using BWA-MEM (*19*). Single nucleotide polymorphisms (SNPs) were identified by using GATK2 (*20*) in unified genotyper mode. Genome positions that had a high quality SNP (>90% consensus, minimum depth ×10, GQ>30) in >1 isolate were extracted. We used pseudosequences of polymorphic positions to create maximum-likelihood trees by using RAxML (The Exelixis Lab, Heidelberg, Germany) (*21*) and calculated pairwise SNP distances between each pseudosequence. We deposited FASTQ sequences in the National Center for Biotechnology Information Short Read Archive under the BioProject PRJNA248042.

### Environmental

Environmental health officers inspected both restaurants and collected food and environmental samples. The food items sampled from the restaurant in outbreak A were targeted on the basis of food histories from initial case-patients and included brown rice with chickpeas, chicken curry, spicy chicken dish with bullet chili peppers, sauces, and salad items. Environmental samples were taken from cutting boards, blenders, water, and taps. No specific food samples remained from the wedding party in outbreak B, so samples were taken from the restaurant. Samples of food items similar to those from outbreak A were collected and included mixed salad, fresh coriander, carrot topping, green chutney, and fresh green chili peppers. Environmental samples were taken from salad tongs, a tea towel, a cutting board and knife used in salad preparation, a blender, and a hot water tap.

We initially sent all food and environmental samples to the PHE Food, Water and Environment laboratory in York to test for enteric pathogens. *E. coli*–positive isolates were then sent to the GBRU for PCR testing for *ipaH*, culture and serotyping.

Environmental health officers investigated food handlers working at the restaurants and in the food supply chain by interviewing the restaurants’ proprietors. Details from identified food suppliers were used to trace the source of the food items and to identify any commonality between the restaurants.

## Results

### Epidemiologic

#### Outbreak A

For outbreak A, PHE was notified of 142 persons with gastrointestinal illness; 108 (76%) were successfully interviewed, resulting in 19 confirmed cases, 88 probable cases, and 1 excluded case due to foreign travel. We recruited and interviewed 28 controls.

**Figure 1:**
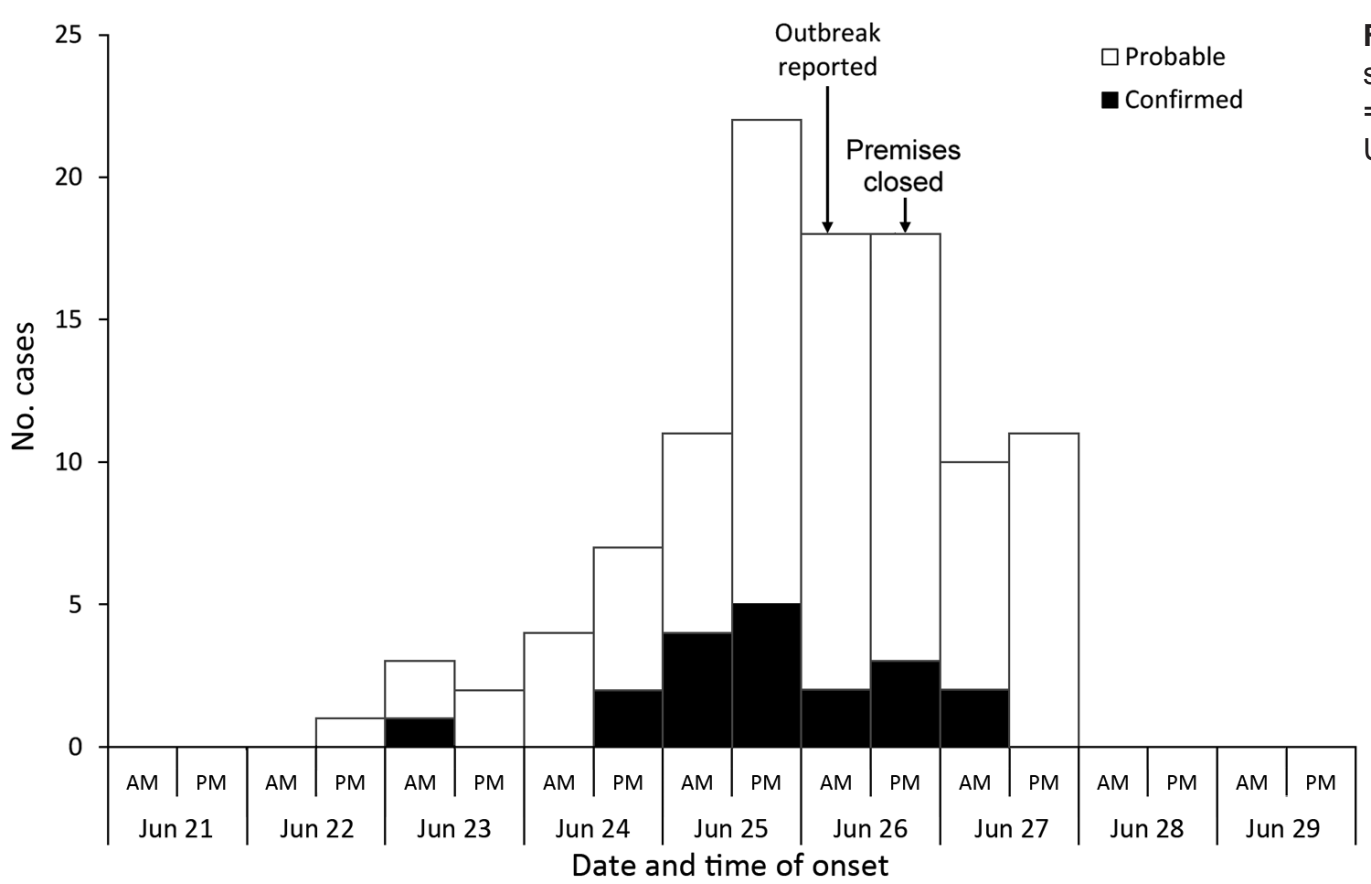
Distribution of cases by symptom onset and case status (n = 107), outbreak A, Nottingham, UK, June 2014.

The onset of symptoms for case-patients ranged from the evening of June 22 to the evening of June 27; peak onset occurred on June 26 (Figure 1). Case-patients reported having eaten in or eaten takeaway from the restaurant during June 18–26 (premises closed on the evening of June 26). Among those with available information (n = 85), the median incubation period was 24 hours (interquartile range [IQR] 17–35, range 6–168 hours). The median age of case-patients was 30 years (IQR 15–39, range 1–75 years); 56 (52%) of the case-patients were male.

The sex of controls and the dates that controls reported eating from the restaurant were the same as those of case-patients. However, controls were significantly younger (p = 0.038), at a median age of 19 years (IQR 8–33, range <1–63 years).

Most (n = 106, 99%) case-patients reported having diarrhea plus a combination of other symptoms. A total of 55 (51.4%) case-patients sought healthcare from general practice medical doctors; in addition, 21 case-patients sought care in a hospital (19.6%), of whom 14 were admitted. When interviewed again ≈30 days after onset of illness, 3 case-patients were still symptomatic. Among case-patients who recovered and whose information was available (n = 87), the median duration of illness was 7 days (IQR 3–10, range 1–21 days).

All items from the restaurant menu were included in the univariable analysis (n = 71). Food items with the highest percentage of case-patients exposed were lettuce (80.4%), cucumber (74.8%), tomatoes (71.0%), and onions (68.2%). Univariable analysis showed that consumption of any of these 4 salad items was positively associated with being a case-patient. A total of 11 food items were included in the multivariable model, but only consumption of lettuce remained a statistically significant risk factor (Table 1). Case-patients were 5 times more likely to have consumed lettuce than were controls (OR 4.99, 95% CI 2.01–12.42). Consumption of lamb donner, a ground meat comprising cuts from various parts of the lamb, also remained in the model but was negatively associated with being a case-patient (OR 0.35, 95% CI 0.14–0.90).

**Table 1:** Multivariable model of exposures associated with EIEC outbreak A, Nottingham, United Kingdom, June 2014*

Exposure	Odds ratio	95% CI	p value
Lettuce	4.99	2.01–12.42	0.001
Lamb donner	0.35	0.14–0.90	0.030
*EIEC, enteroinvasive *Escherichia coli.*			

#### Outbreak B

From a list of 60 persons who attended the wedding, we obtained information related to outbreak B for 41 (68%). Of those, 15 persons met the outbreak case definition (3 confirmed and 12 probable cases), 24 had no signs or symptoms of illness, and 2 were excluded because they did not consume food at the wedding. The median age of case-patients was 34 years (IQR 12–36, range 3–64 years); 10 (67%) were male.

**Figure 2:**
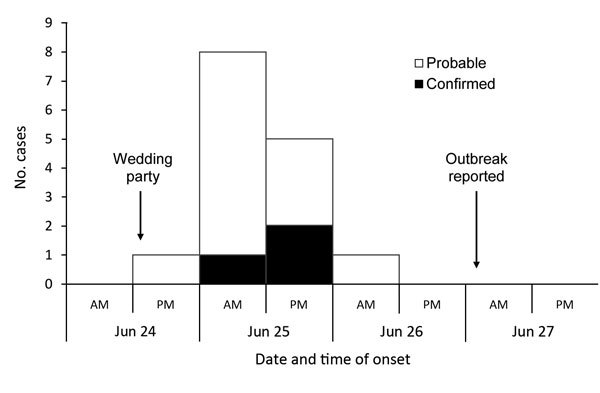
Distribution of cases by symptom onset and case status (n = 15), outbreak B, Nottingham, UK, June 2014.

The symptom onset date ranged from the evening of June 24 to the morning of June 26; peak onset was on the morning of June 25 (Figure 2). Among those for whom information was available, the median incubation period was 11 hours (IQR 10–19, range 9–37 hours) (Table 2), which was significantly shorter than the incubation period in outbreak A (p = 0.002). 

**Table 2:** Characteristics of case-patients from EIEC outbreaks A and B, Nottingham, United Kingdom, June 2014*

Characteristics	Outbreak A, n = 107	Outbreak B, n = 15
Male sex, %	52	67
Median age, y (IQR)	30 (15–39)	34 (12–36)
Dates exposed	June 18–26	June 24
Onset dates	June 22–27	June 24–26
Median incubation period, hours (IQR)	24 (17–34)	11 (10–19)
Contacted GP, no. (%)	55 (51.4)	8 (53)
Contacted hospital, no. (%)	21 (19.6)	0 (0)
Median duration of illness, d (IQR)	7 (3–10)	4 (2–10)
*EIEC, enteroinvasive *Escherichia coli*; GP, general practice medical doctor; IQR, interquartile range.		

All case-patients reported diarrhea plus a combination of other symptoms. A total of 8 (53%) case-patients sought healthcare for their illness from their general practitioner, but none was admitted to a hospital. Among recovered case-patients whose information was available (n = 11), the median duration of illness was 4 days (IQR 2–10, range 2–25 days). When interviewed ≈30 days after onset of illness, 1 case-patient was still symptomatic.

The overall attack rate varied by sex: male patients were ≈2 times more likely to have a case than were female patients (ratio [RR] 2.33, 95% CI 0.98–5.57, p = 0.042). However, because of the small size of the cohort, it was not possible to meaningfully stratify the analysis by sex.

Univariable analysis showed that drinking tap water was positively associated with being a case-patient (RR 2.29, 95% CI 1.06–4.91), whereas lentil curry was negatively associated (RR 0.21, 95% CI 0.03–1.38). Of the 7 menu items included in the multivariable model, 6 were independently associated with being a case-patient (Table 3). Multivariable analysis showed the risk for illness was ≈5 times higher among those who ate salad (RR 4.79, 95% CI 1.97–11.62), 6 times higher among those who drank tap water (RR 5.73, 95% CI 1.85–17.76), and 4 times higher among those who ate chicken curry (RR 3.94, 95% CI 1.52–10.19) compared with those who did not consume these items. The consumption of naan bread (RR 0.16, 95% CI 0.05–0.51), milk pudding (RR 0.36, 95% CI 0.14–0.90), or green chutney (RR 0.26, 95% CI 0.77–0.86) was negatively associated with illness.

**Table 3:** Multivariable model of exposures associated with EIEC outbreak B, Nottingham, United Kingdom, June 2014*

Exposure	Risk ratio	95% CI	p value
Salad	4.79	1.97–11.62	0.001
Tap water	5.73	1.85–17.76	0.003
Naan bread	0.16	0.05–0.51	0.002
Milk pudding	0.36	0.14–0.90	0.029
Chicken curry	3.94	1.52–10.19	0.005
Green chutney	0.26	0.77–0.86	0.027
*EIEC, enteroinvasive *Escherichia coli.*			

### Microbiological

Fecal samples from 44 case-patients and 17 food handlers in outbreaks A and B were submitted for microbiological testing (Table 4). Across both outbreaks, EIEC O96:H19 was isolated from 23 case-patient samples, and the *ipaH* gene was detected in samples from 14 other case-patients; 2 case-patients from outbreak B also tested positive for *Campylobacter jejuni* by multiplex PCR.

**Table 4:** Summary of EIEC fecal sample test results by outbreak, Nottingham, United Kingdom, June 2014 (n = 61)*

Fecal sample test and result	Outbreak A, no. (%)			Outbreak B, no. (%)	
	Case-patients	Food handlers		Case-patients	Food handlers
EIEC O96:H19, culture positive	20 (57.1)	4 (33.3)		3 (33.3)	0
EIEC PCR positive, *ipaH* gene	9 (25.7)	5 (41.7)		5 (55.6)	0
EIEC-negative, PCR and culture	6 (17.1)	3 (25.0)		0	5 (100)
Leaked sample not processed	0	0		1 (11.1)	0
Total samples tested	35 (100)	12 (100)		9 (100)	5 (100)
*EIEC, enteroinvasive *Escherichia coli*.					

Fecal samples from all 12 food handlers in outbreak A were tested; 4 were culture-positive for EIEC O96:H19. All 4 persons were asymptomatic, but 1 reported travel to Pakistan during May 2014 and was ill for 3 days on return to the United Kingdom. The *ipaH* gene was detected in samples from 5 food handlers, of whom 2 were symptomatic, with onset dates of June 25 and 26, 2014. Samples from 2 food handlers who were PCR-positive for EIEC tested positive for verocytotoxin-producing *Escherichia* *coli* by using multiplex PCR. Of 6 food handlers in outbreak B, samples from 5 were tested and were negative for EIEC.

### Environmental

A total of 41 food and environmental samples taken from the 2 restaurants were sent to the GBRU. Of these, EIEC O96:H19 was isolated from 1 lettuce sample taken from the restaurant in outbreak A, which was the only lettuce sample taken from the restaurant. The lettuce had been washed, cut, and then stored in a container in a chilled display unit. No other organisms were detected by multiplex PCR from these samples. 

Inspections of the restaurant in outbreak A identified potential opportunities for cross-contamination between raw meats and ready-to-eat foods during storage, washing, and cooking; chilled food items being stored above the temperature required by law; and inadequate handwashing facilities and practices. No commonalities were identified among food handlers, the food suppliers, or brands of lettuce in the 2 restaurants.

### Whole Genome Sequencing

We sequenced 9 isolates from samples in outbreaks A and B: from 4 case-patients, 1 food handler, and the lettuce from outbreak A, and from 3 case-patients in outbreak B. Phylogenetic analysis showed that all isolates from case-patients and the food handler were either identical or differed by a single SNP from that sequenced from the lettuce sample.

## Discussion

We describe investigations into 2 outbreaks of EIEC infections that affected 157 persons in Nottingham, UK. The epidemic curves were indicative of 2 common-source outbreaks linked to a restaurant and a wedding party in another restaurant within 0.1 miles of one another. Although whole-genome sequencing showed that the organisms isolated from case-patients in both outbreaks were genetically related, no specific epidemiologic link was identified.

In Europe, reports of EIEC outbreaks have previously been uncommon. However, these 2 large outbreaks and the 2012 outbreak in Italy (*5*) suggest a possible undocumented increase in this pathogen in Europe. Analyses of isolates from these outbreaks plus a sporadic case in Spain found all to be the rare serotype O96:H19 and belong to an EIEC clone not seen before the 2012 outbreak in Italy (*22*).

Difficulties surround the surveillance and diagnosis of EIEC, possibly resulting in underreporting. Clinicians, as well as pathologists based in laboratories, may be unaware of EIEC as a pathogen for diarrheal illness, especially when case-patients appear to have acquired their infection within the United Kingdom, and frontline diagnostic tests are not usually able to distinguish EIEC from nonpathogenic *E. coli* (*5*). In England, the prevalence of this organism is currently unknown. An intestinal infectious disease study in England during 1993–1996 did not identify any cases of EIEC (*23*), but it was not tested for in a repeat study during 2008–2009, so it is unknown if this status remained unchanged (*24*). In the outbreaks we investigated, the prompt notification and referral of samples to the reference laboratory enabled us to quickly identify and microbiologically confirm EIEC in several cases. The symptom profile and incubation period of cases from outbreaks A and B are consistent with those reported for EIEC (*5*,*25*). Based on the proportion of case-patients admitted to hospitals, it appears that case-patients in outbreak A experienced more severe illness than those in outbreak B; however, the reason for this is unknown.

In outbreak A, the combined epidemiologic, microbiological, and environmental findings implicated lettuce as the vehicle of infection. Lettuce and other salad items requested were either served directly onto the food or were placed in a small plastic bag to accompany takeaway dishes. EIEC foodborne outbreaks have previously been documented (*26*–*28*), and in an outbreak in Italy, EIEC infection was found to be associated with vegetables, although EIEC was not isolated from the food (*5*).

The source of the organism in this outbreak is less clear: of the 12 food handlers associated with outbreak A, 9 (75%) tested positive for EIEC, but most reported they were asymptomatic, so we are unable to ascertain how or when they acquired their infection. However, 1 food handler who was asymptomatic at the time of the outbreaks but who tested positive for EIEC reported becoming ill with gastrointestinal symptoms on return from Pakistan in May 2014. Although the food handler reported not working while symptomatic, there have been reports in the literature of asymptomatic persons shedding EIEC up to 1 year after infection (*25*), so it is plausible that this food handler may have introduced the organism into the restaurant. Poor food hygiene standards identified at the restaurant may have facilitated cross-contamination among the other food handlers through person-to-person transmission or consumption of contaminated food items.

A second hypothesis for the source of infection is that contaminated lettuce was introduced into the restaurant. However, we found no commonality with the lettuce supplier for outbreaks A and B, and we were not notified of any further outbreaks of EIEC, which might have been expected if there was an issue with the supplier. Considering the challenges in diagnosis and surveillance of EIEC detailed above, isolated cases that were not part of a localized cluster would have been difficult to identify.

The choice of case-nominated controls may have introduced selection bias to our study. Our assessment showed that controls were significantly younger than case-patients, and the high attack rate among those who ate at the restaurant resulted in only a small number of suitable controls being identified. The restaurant had no daily records of customers; therefore, the choice of case-nominated controls was the most pragmatic and timely way of recruiting controls. Power and sample size calculations showed that our study was adequately powered to detect lettuce as a vehicle of infection, but any food items with smaller effect sizes may not have been identified. However, we believe our epidemiologic findings are valid because they are supported by environmental and microbiological findings.

For outbreak B, we were unable to identify a definite source and route of EIEC infection at the wedding party. Power calculations found the study to be underpowered, and we did not have any microbiological evidence to identify the true source or vehicle of infection. Salad was a food item associated with the risk for illness, but no links could be found between the 2 restaurants related to food handlers, customers, or suppliers, despite its close proximity to the restaurant in outbreak A. Some wedding party guests chose not to participate in the study; therefore, the study cohort may not be representative of the outbreak cohort.

Prompt control measures seemed to be effective in limiting further transmission of EIEC. Outbreak A stopped after the restaurant was closed, and in outbreak B, no cases were identified outside of the wedding party. We found little in the literature on the management of EIEC cases to prevent secondary transmission. In both outbreaks, guidelines for preventing *Shigella* infections (*29*) were used because of the genetic similarity of EIEC to *Shigella*. Case-patients and contacts in high risk groups were excluded from working or attending high-risk settings such as eating establishments, day nurseries, and healthcare facilities until microbiological clearance, defined as 2 negative fecal specimens taken at intervals of not less than 48 hours, had been achieved. Case-patients who were not in a high-risk group were provided with an information sheet detailing advice on enteric precautions they should take to prevent the spread of the infection. 

These 2 outbreaks of EIEC in Nottingham during June 2014 were uncommon for England, but highlight that EIEC has the capacity to cause large and potentially severe gastrointestinal outbreaks in Europe and should be considered as a potential pathogen in foodborne outbreaks.
